# Evaluation of the safety and efficacy of concentrated growth factors for hair growth promotion in androgenetic alopecia patients: A retrospective single‐centre, single‐arm study

**DOI:** 10.1111/jocd.16519

**Published:** 2024-08-21

**Authors:** Saisai Cao, Mengyi Zhu, Ye Bi

**Affiliations:** ^1^ Department of Plastic Surgery Peking University People's Hospital Beijing China; ^2^ Department of Dermatology Peking University People's Hospital Beijing China

**Keywords:** androgenetic alopecia, concentrated growth factor, hair loss

## Abstract

**Objective:**

This study was designed to evaluate the safety and efficacy of injections of concentrated growth factors (CGF) for hair growth promotion in androgenetic alopecia (AGA) patients.

**Methods:**

A retrospective review of AGA patients treated with injections of CGF at our center from October 2021 to April 2023 was performed. A total of 3 injections were administered every 3–4 weeks apart, and evaluation were performed before the first injection and at 3 months, 6 months later. The outcomes were assessed by trichoscopy photomicrographs and the Global Aesthetic Improvement Scale (GAIS).

**Results:**

At 3 months after the first injection, the hair density and hair growth ratio were significantly improved. Significant improvements were found in GAIS score by both patients and independent doctors and the hair growth promotion was sustained for 6 months after first treatment.

**Conclusions:**

According to this tiny single‐arm trial, the use of CGF injection may help AGA by increasing terminal hair density and hair density. No severe topical or systemic adverse events occurred during the treatment.

## 1 INTRODUCTION

Androgen‐related progressive hair loss, known as androgenetic alopecia (AGA), affects both men and women, particularly those with a family history of the condition, and worsens with aging, it is characterized by progressive hair loss, particularly of scalp hair.^1^, ^2^ The prevalence of AGA differs by race and gender, the incidence are up to 21.3% among males and 6.0% among females in China, and the incidence is much higher in Caucasians with up to 80% of men and 40% of women by the age of 70 or beyond.^3^, ^4^ As the most frequent long‐term concern that plastic surgeons and dermatologists see on a global basis, the impact of AGA affects the physical appearance and psychosocial experiences of individuals and evokes emotional distress.^5^

Despite AGA has been treated for decades, adverse reactions or low efficiency appear to be barriers to successful controlling and treatment.^6^ Various options are available, including follicular unit transplantation (FUT) and medications. The former is limited because of invasiveness, pain, and bleeding as a surgical procedure, while the latter includes oral finasteride and topical minoxidil.^2^ Besides, over the past decade, there has been a growing utilization of platelet‐rich plasma (PRP) as an autogenous solution derived from blood to treat AGA. This solution is known for its elevated levels of platelets and growth factors (GFs).^7^, ^8^ Concentrated Growth Factors (CGF) is an advanced version of PRP with higher levels of GFs due to its varied centrifugation process.^9^ It showed a distinct potential in wound healing and dentistry in recent studies.^10^, ^11^ However, only a few studies evaluated CGF injection in AGA patients to improve hair density and reduce the appearance of hair loss.^12^

We believe that the injections of CGF are both safe and effective for the treatment of AGA and hope to verify this finding in this study.

## 2 METHOD

This was a retrospective study of AGA patients treated with CGF at our center from October 2021 to April 2023. The study protocol was approved by the Ethics Committee in accordance with the Declaration of Helsinki, and written informed consent was obtained from all study participants. The study excluded patients who had undergone anti‐AGA treatment within the past 6 months or had a previous hair transplantation surgery. The inclusion criteria were met by 15 consecutive patients and no one was excluded from the study. Their medical records were then reviewed.

### 2.1 CGF preparation and injection

In this study, the CGF was produced as follows: each patient had 18–27 mL of whole blood drawn depending on the hair loss area. Each sterile serum blood collection tube (Vacuette, Greiner Bio‐One, Kremsmünster, Austria) was injected with a 9 mL venous blood sample to produce CGF. The sample was promptly subjected to centrifugation using a special machine with programmed varying speeds (Medifuge CGF MF200100 Silfradent S.R.L., Sofia, FC, Italy).^13^ After centrifugation, the blood was separated into three different layers. The bottom layer consisted of red blood cells, the top layer contained platelet‐containing plasma, and the middle layer was known as the buffy coat, which contained white leukocytes. After that, 2 mL of plasma supernatant was removed and discarded, and the remaining plasma was collected to produce approximately 2–3 mL of CGF from every 9 mL of whole blood.

Before injection, we used compound lidocaine cream (Tongfang Pharmaceutical Co., Ltd., Beijing, China) for anesthesia. The cream contained 25 mg of procaine and 25 mg of lidocaine per gram and was evenly applied to the surface of the treatment area 30–45 min before the treatment. The CGF was injected subcutaneously using a 34‐gauge needle in the hairless area by hand. The amount injected was 0.05 mL/cm^2^. The injection was repeated two more times every 3–4 weeks.

### 2.2 Efficacy assessment

The patients were assessed at baseline, 3 months (about 1 month after the last injection), and half a year after the first injection. A trichophotograph was taken of the treated areas using a dermatoscope (DL200 Hybrid; Dermlite, San Juan Capistrano, CA) to measure the hair count. Hair was classified into terminal hair (long, thick, pigmented) and vellus hair (thin, unpigmented) based on its morphology. Hair densities (counts/cm^2^) of both terminal hair and vellus hair were calculated for statistical analysis, and the T/V ratio was determined in the treatment areas. We compared the hair density at each visit to the baseline to calculate the HG ratio. In the meantime, an independent doctor compared all photographs with the baseline photos using the Global Aesthetic Improvement Scale (GAIS). Additionally, patients self‐evaluated using a satisfaction survey.(−1: significantly worse hair loss than before treatment; 0: no change in hair loss; 1: slight improvement of hair loss; 2: significant improvement of hair loss but a recommendation for further treatment to obtain a better hair appearance; 3: significant improvement of hair loss with an optimal hair appearance and minimal further treatment requirements).

Dichotomous variables were expressed as proportions, whereas continuous variables were expressed as mean ± SD or median and interquartile range (IQR) (25–75th percentiles). Within‐group differences during follow‐up compared with baseline and between‐group differences were evaluated using a paired *t*‐test. A *p*‐value <0.05 indicates that the difference was statistically significant. All analyses were performed using the SPSS 24.0 software (SPSS Inc., Chicago, IL, United States).

## 3 RESULTS

**FIGURE 1:**
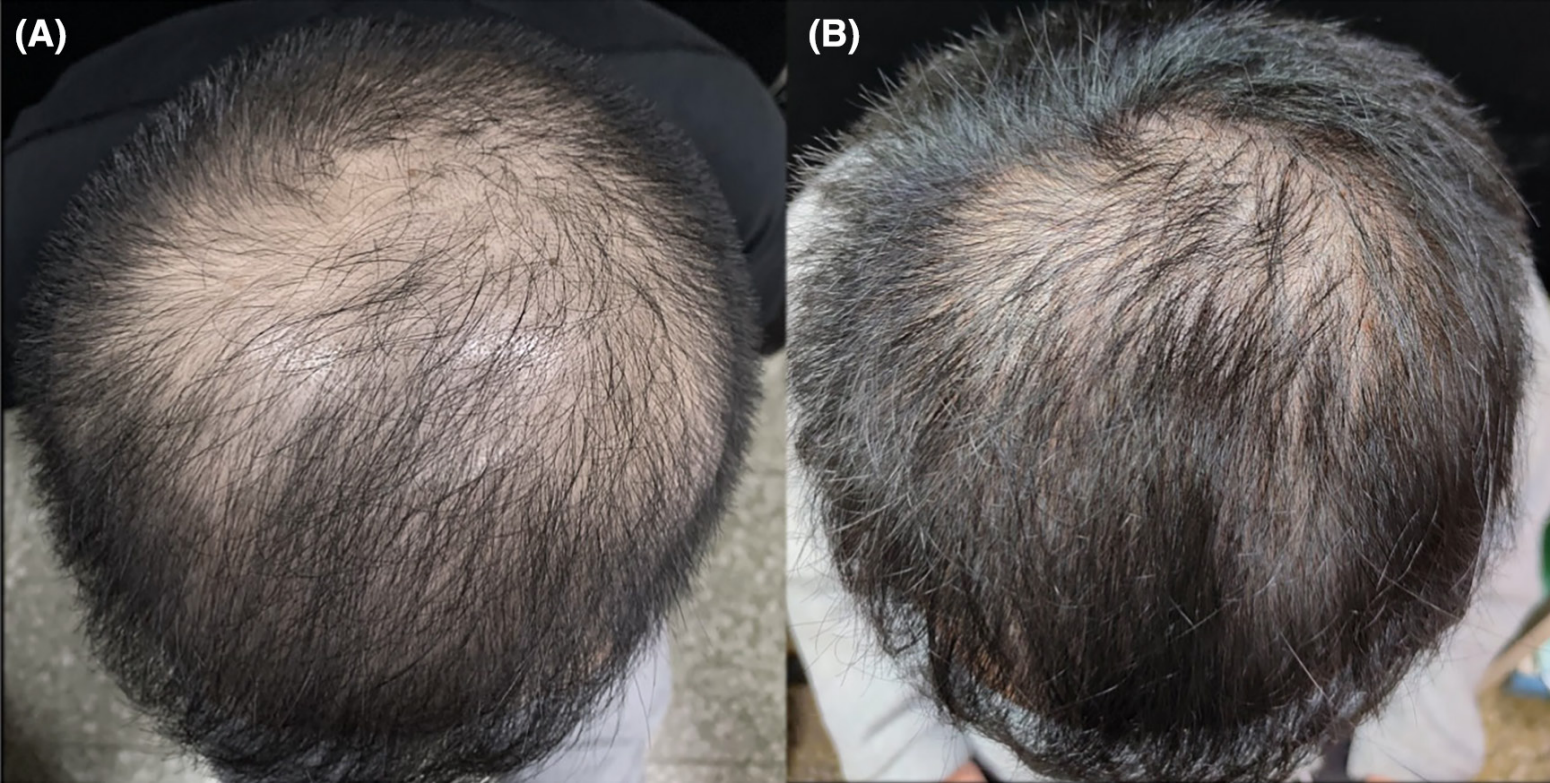
A 30‐year‐old male patient with BASP V2 AGA (case 1) (A) before treatment and (B) 6 months after first treatment.

**FIGURE 2:**
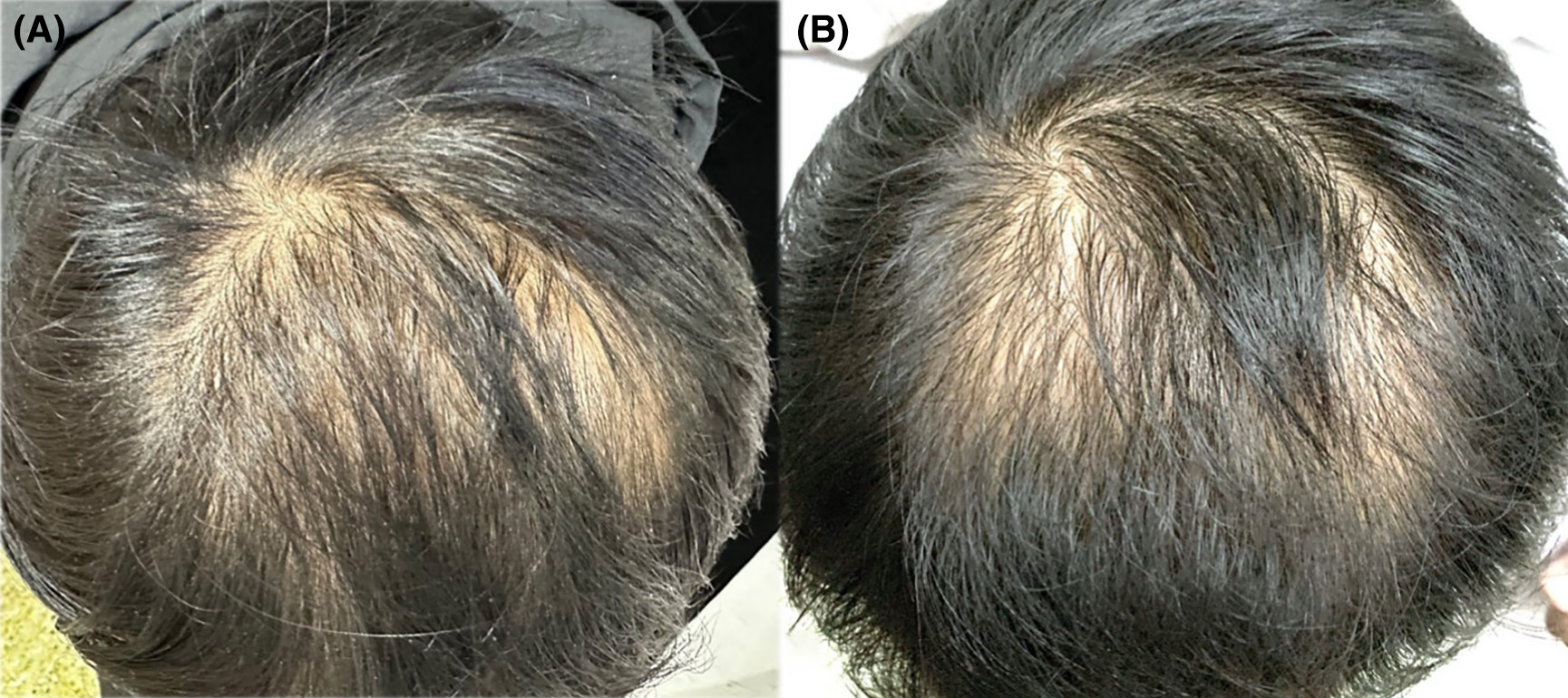
A 32‐year‐old male patient with BASP M1V1 AGA (case 2) (A) before treatment and (B) 6 months after first treatment.

A total of 15 AGA patients (2 females and 13 males) were enrolled in the study. The basic characteristics are shown in Table 1. The median age was 31 years old (IQR, 24–34 years old), and the history of hair loss among the subjects was 1–8 years. Five patients used topical minoxidil for the treatment of alopecia previously. Representative images of patients before and 6 months later after the first injection are shown in Figures 1 and 2.

**TABLE 1:** Characteristics of the study patients.

Patient number	Sex/age (years)	The history of AGA (years)	Hair loss type (BASP)	Previous treatment
(1)	M/30	2	V2	Topical minoxidil
(2)	M/32	5	M1V1	None
(3)	M/28	3	V2	None
(4)	M/51	8	V2	Topical minoxidil
(5)	M/32	1	M1V2	None
(6)	F/33	3	F1	None
(7)	M/41	5	M1V1	None
(8)	M/25	3	M1V1	None
(9)	M/32	5	M2V1	Topical minoxidil
(10)	M/27	3	V2	None
(11)	M/31	2	M1V1	None
(12)	M/24	5	M1V1	Topical minoxidil
(13)	M/40	7	F3	Topical minoxidil
(14)	M/34	3	M2V1	None
(15)	F/40	2	F1	None

Abbreviations: BASP, basic and specific classification; F, female; M, male.

Table 2 shows relevant changes in hair growth parameters, and GAIS of physicians and patients. After 12 weeks of treatment, the total mean hair density showed a significant increase when comparing the photographs taken before. The mean hair density increased by about 40 (per hairs/cm^2^) compared to baseline (*p* < 0.01). In terms of changes in terminal hair showed a sustained increase after treatment (*p* < 0.01). Regarding the hair growth ratio, it was 1.54 ± 0.10 at the end of follow‐up (*p* < 0.01). However, no difference was found in T/V radio in both 3 months and 6 months. The patients ranked the mean GAIS score as 0.8 ± 0.77 at 3 months and 1.93 ± 0.88 at 6 months (*p* < 0.01), and by independent doctors that was 0.47 ± 0.64 and 1.67 ± 0.72 (*p* < 0.01), both showed this complex treatment was satisfactory and sustainable. Especially, none of the patients showed any deterioration compared to their baseline condition. In addition, three patients (20%) reported a reduction in greasy hair texture, itching, or burning sensations after 1–2 weeks since the combined treatment started.

**TABLE 2:** Relevant change of hair growth parameters, GAIS of physicians and patients.

Hair parameter		Baseline	3 Months	6 Months	*p* ^a^	*p* ^b^
	Hair density (per hairs/cm^2^)	81.1 ± 16.2	101.8 ± 23.8	125.5 ± 27.1	0.01	<0.01
	Terminal hair density (per hairs/cm^2^)	51.8 ± 16.0	63.2 ± 18.2	84.2 ± 22.6	0.08	<0.01
	Vellus hair density (per hairs/cm^2^)	31.2 ± 4.7	40.4 ± 8.6	44.1 ± 7.7	<0.01	<0.01
	T/V radio	1.70 ± 0.60	1.59 ± 0.47	1.91 ± 0.45	0.59	0.29
	Hair growth radio(index)	1.00 ± 0.00	1.25 ± 0.12	1.54 ± 0.10	<0.01	<0.01
**GAIS (physicians)**					***p***	
	−1 (*n*/%)	‐	0 (0)	0 (0)	‐	
	0 (*n*/%)	‐	9 (60)	1 (6.7)	‐	
	1 (*n*/%)	‐	5 (33.3)	4 (26.7)	‐	
	2 (*n*/%)	‐	1 (6.7)	9 (60)	‐	
	3 (*n*/%)	‐	0 (0)	1 (6.7)	‐	
	Average GAIS		0.47 ± 0.64	1.67 ± 0.72	< 0.01	
**GAIS (patients)**					***p***	
	−1 (*n*/%)	‐	0 (0%)	0 (0%)	‐	
	0 (*n*/%)	‐	6 (40)	1 (6.7)	‐	
	1 (*n*/%)	‐	6 (40)	3 (20)	‐	
	2 (*n*/%)	‐	3 (20)	7 (46.7)	‐	
	3 (*n*/%)	‐	0 (0)	4 (26.7)	‐	
	Average GAIS		0.8 ± 0.77	1.93 ± 0.88	< 0.01	

^a^ Denotes comparisons between 3 months and baseline.

^b^ Denotes comparisons between 6 months and baseline; GAIS: Global Aesthetic Improvement Scale.

No patient reported any adverse effects such as pain, redness, or bleeding during or after the injection. Moreover, there were no major side effects during the treatment.

## 4 DISCUSSION

Androgenic alopecia is the most common and sex‐hormone mediated form of alopecia, affecting both men and women.^4^, ^14^ Patients afflicted with AGA undergo significant impairment of quality of life caused by a progressive reduction in the diameter, length, and pigmentation of the hair.^15^ The development of AGA is primarily caused by dihydrotestosterone (DHT), a hormone that affects hair follicles sensitive to androgens.^16^ The enzyme 5α‐reductase (5‐AR) is responsible for converting testosterone to DHT. Subsequently, DHT binds to hair follicle androgen receptors to activate signaling pathways that cause miniaturization and eventual hair loss.^17^, ^18^

AGA can be treated in several ways, but it is still challenging due to adverse reactions or low efficiency. Surgical techniques have been refined over a century, both FUT and follicular unit excision (FUE) can provide satisfactory results, but the success of the procedure depends on selecting the appropriate patient. However, both methods are invasive and can cause pain and bleeding. Additionally, the survival of the transplanted follicle units is most dependent on the surgeon's expertise and technical skills, as well as overall resources.^19^

Nonsurgical treatments and medications are available for AGA,

Nonsurgical treatment, including lifestyle adjustments and medications, but only two FDA‐approved drugs exist: oral finasteride and topical minoxidil.^2^ Topical minoxidil (2% or 5%) promotes hair growth by improving nutrient delivery to hair follicles through dilating scalp blood vessels.^20^ However, minoxidil is unsatisfied with only nearly 40% efficacy with topical side effects including hypertrichosis in the face and temporary telogen.^21^, ^22^ Therefore, several combination therapies were explored for superior efficiency, including adding low‐level light therapy to topical minoxidil therapy and adding other herbal solutions to topical minoxidil therapy.^22^, ^23^ Both of these attempts showed highly efficacious as compared to topical minoxidil alone. Another systematic review showed the use of microneedling in conjunction with topical minoxidil can enhance hair growth, increase hair diameter, reduce hair loss, and decrease side effects compared to using minoxidil alone.^24^

More importantly, it has been demonstrated that various GFs including vascular endothelial growth factor (VEGF) and epidermal growth factor (EGF) improve follicle vascularization to promote hair growth and increase hair follicle and hair size.^25^ Different autogenous solutions like PRP or adipose tissue‐derived stem cell conditioned media (ADSC‐CM) have been used to treat AGA in increasing numbers over the last decade.^8^, ^26^ As the PRP and its secretory factors were considered to contribute to the regulation of hair growth, CGF may be considered as an upgraded version of PRP and was extracted with a variable speed centrifugation prepared by drawing venous blood.^11^ However, only a few studies mentioned the use of CGF for hair growth. Steward studied 20 males treated with PRP injection and CGF gel applied topically after microneedling, this study demonstrated that the administration of PRP and CGF has a positive effect on AGA without significant side effects, and the hair count raised about 30%.^27^ After that, a Chinese randomized controlled clinical trial showed similar results, three CGF injections were administered to half of the scalp, while the other side received a placebo and minoxidil was applied twice daily on both sides in 16 AGA patients.^12^ The CGF group showed better hair growth and significant improvements in both hair density and proportion of terminal hair, the hair density increased by over 140% and T/V radio increased by over 50%. In our study, the use of CGF injection alone also significantly promoted hair growth in hair density and terminal hair density, the improvement of hair density was better than PRP injection combined with CGF gel applied topically.^27^ However, the combination of CGF injection and topical minoxidil showed more hair growth and better GAIS in both patient and physician assessments compared to our study.^12^ On the other hand, 20% of patients in our study reported a reduction in greasiness of hair and other discomfort of the scalp. No systematic side effects occurred.

However, the specific mechanisms of CGF in the treatment of AGA are unclear. Previous study demonstrated that the AGA hair follicles have a deficiency of CD34+ hair follicle progenitor cells and the CD34+ stem cells promote hair growth.^28^, ^29^ On the other hand, CGF releases GFs from the adhered platelets and fibrin concentrate. Other studies found that there are a high number of CD34‐positive cells in the CGF layer.^30^ These findings may explain the mechanisms of CGF contributed to the treatment of AGA. Besides, CGF has an advantage in providing steady clinical application products. There is variability in the preparation of PRP due to differences in platelet concentration standardization and protocols for centrifugation and activators in various medical centers.^31^ CGF preparation is simple with single‐spin centrifugation and no need for additional manipulations. This treatment is faster as the production of CGF takes only approximately 15 min. These advantages are conducive to its promotion and application in the future.

It is worth mentioning that nerve block anesthesia of supraorbital and supratrochlear was used before injection in most studies.^12^, ^27^ However, we only used compound lidocaine cream for the anesthesia of the scalp, and a 34G needle was applied, with no patients reported pain during the injection process. The advantage may be due to the use of a thinner needle as the injection was performed with a 34‐gauge needle.

### 4.1 Limitations

Our study has several notable limitations. This study is inherently subject to a relatively small number of patients enrolled, and the participants enrolled in this research may or may not represent AGA patients in the general population. Second, the lack of a control group also limits the importance of our findings. Finally, a long‐term follow‐up period is needed to study the relapses of hair loss after treatment.

## 5 CONCLUSION

The use of CGF injection significantly improved hair density, terminal hair density, and GAIS. There were no severe topical or systemic side effects during the treatment. We believe that this complex therapy could be a promising therapy for AGA. However, to confirm the effectiveness and safety of CGF on hair growth and explain the mechanisms responsible, further research with larger sample sizes may be required.

## AUTHOR CONTRIBUTIONS

SC and YB contributed to the conception and study design. SC and CW contributed to writing the article. SC helped in the data collection, analysis, and interpretation. All authors contributed to the article and approved the submitted version.

## FUNDING INFORMATION

No funding.

## CONFLICT OF INTEREST STATEMENT

The authors declare that the research was conducted in the absence of any commercial or financial relationships that could be construed as a potential conflict of interest.

## ETHICS STATEMENT

All procedures performed in studies involving human participants were in accordance with the ethical standards of the 1964 Helsinki Declaration and its later amendments or comparable ethical standards.

## CONSENT

All patients signed the informed consent form after understanding the nature of the trial.

## Data Availability

The data that support the findings of this study are available from the corresponding author upon reasonable request.
